# Lysyl oxidase-like 2 inhibition ameliorates glomerulosclerosis and albuminuria in diabetic nephropathy

**DOI:** 10.1038/s41598-018-27462-6

**Published:** 2018-06-21

**Authors:** Stefanie Stangenberg, Sonia Saad, Heidi C. Schilter, Amgad Zaky, Anthony Gill, Carol A. Pollock, Muh Geot Wong

**Affiliations:** 1 Renal Research, Kolling Institute, Northern Sydney Local Health District, St Leonards, NSW Sydney, Australia; 2 Sydney Medical School Northern, University of Sydney, NSW Sydney, Australia; 3 Pharmaxis Pharmaceutical Ltd., Frenchs Forest, NSW Sydney, Australia; 4 Department of Cancer Research and Pathology Kolling Institute, Northern Sydney Local Health District, St Leonards, NSW Sydney, Australia

## Abstract

Diabetic nephropathy is characterised by the excessive amount of extracellular matrix in glomeruli and tubulointerstitial space. Lysyl oxidase-like 2 (LOXL2) is elevated in renal fibrosis and known to play key roles in ECM stabilisation by facilitating collagen cross-links, epithelial to mesenchymal transition and myofibroblast activation. Thus, targeting LOXL2 may prove to be a useful strategy to prevent diabetic nephropathy. We explored the renoprotective effect of a selective small molecule LOXL2 inhibitor (PXS-S2B) in a streptozotocin-induced diabetes model. Diabetic mice were treated with PXS-S2B for 24 weeks and outcomes compared with untreated diabetic mice and with telmisartan treated animals as comparator of current standard of care. Diabetic mice had albuminuria, higher glomerulosclerosis scores, upregulation of fibrosis markers and increased renal cortical LOXL2 expression. Treatment with PXS-S2B reduced albuminuria and ameliorated glomerulosclerosis. This was associated with reduced expression of glomerular fibronectin and tubulointerstitial collagen I. The renoprotective effects of both PXS-S2B and telmisartan were more marked in the glomerular compartment than in the tubulointerstitial space. The study reveals that LOXL2 inhibition was beneficial in preserving glomerular structure and function. Thus, LOXL2 may be a potential therapeutic target in diabetic nephropathy.

## Introduction

Diabetic nephropathy, clinically characterised by increasing proteinuria and an eventual decline in glomerular filtration rate, is the leading cause of end-stage kidney disease (ESKD) worldwide and the incidence is expected to rise^1^. Current treatments of diabetic nephropathy are limited to blockade of the renin-angiotensin-aldosterone system (RAAS). However, despite RAAS blockade a significant residual risk of progression to ESKD remains^2,3^. In early stages the histopathology is characterised by glomerular basement membrane (GBM) thickening, often paralleled by tubular basement membrane thickening, followed by mesangial expansion, which can lead to nodular and diffuse glomerulosclerosis. Other features of diabetic nephropathy include arteriolar hyalinosis, atrophic tubules and tubulointerstitial fibrosis as a late manifestation^4^. While the various lesions of diabetic nephropathy progress at varying rates, many of the characteristic manifestations are a consequence of extracellular matrix (ECM) accumulation ultimately leading to renal fibrosis.

The extracellular matrix in healthy tissues is a scaffold to maintain tissue integrity and a reservoir for mediators of cell signaling and cell growth^5^. The ECM is a highly dynamic structure that is in constant flux of remodeling through matrix synthesis and degradation. Dysregulation of this tightly regulated balance will lead to exaggerated matrix deposition and an exacerbation of disease progression. Indeed, it has been reported that a fibrotic ECM can perpetuate fibrogenesis by stimulating myofibroblasts to synthesise further ECM proteins in a positive feedback loop^6^. In renal fibrosis ECM becomes the principal component replacing normal renal parenchyma. Targeting mediators of the dysregulated ECM remodeling process may offer a promising therapeutic opportunity for kidney fibrosis.

The lysyl oxidase family is a group of amine oxidases that play a crucial role in ECM remodeling and are foremost known for their ability to promote covalent cross-linking of collagen in the ECM^7^. The five members of the lysyl oxidase family include the originally identified lysyl oxidase (LOX) and four lysyl oxidase-like proteins (LOXL1, LOXL2, LOXL3, LOXL4) that share sequence homology and have similar function. These copper-dependent enzymes catalyze the oxidation of lysine residues in collagen and elastin to highly reactive aldehydes, which spontaneously condense with other lysyl oxidase-derived aldehydes or unmodified lysine residues to generate intermolecular cross-links between the collagen fibers. The cross-linked collagen renders the ECM resistant against proteolytic enzymes and the resulting tissue stiffness is a prerequisite for the activation of myofibroblasts^8^. In addition to its extracellular function of collagen cross-linking, LOXL2 in particular is involved in transcriptional regulation by promoting epithelial-to mesenchymal transition (EMT)^9^. LOXL2 is a downstream-target of hypoxia-inducible factor-1α (HIF-1α)^10^ and is induced by transforming growth factor beta (TGFβ)^11^, both of which are key players in the pathogenesis of tissue fibrosis. Indeed, an increase of LOXL2 expression has been extensively investigated in fibrotic diseases of lung^12,13^ and liver^13,14^. However its role in renal fibrosis is limited to a few studies^10,15^ and has not been explored as a treatment target in kidney disease. PXS-S2B is a novel haloallylamine-derived, small-molecule LOXL2 inhibitor with oral bioavailability, high potency and strong selectivity for LOXL2. The compound’s selectivity for LOXL2 and its characteristics have been previously published^16^. The present study aims to explore the role of LOXL2 in a renal fibrosis model of diabetic nephropathy and examined the therapeutic potential of PXS-S2B as a renoprotective small molecule.

## Results

### Clinical characteristics

Endothelial nitric oxide synthase knockout (eNOS^−/−^) mice were chosen due to their ability to develop accelerated renal injury after streptozotocin (STZ) induced diabetes^17^. The clinical characteristics of the groups are summarised in Table 1. STZ treated mice had elevated fasting blood glucose levels (BGL) (P < 0.01 vs. control (Ctrl)), lower body weight and higher glycated haemoglobin (HbA1c) (P < 0.01 vs. Ctrl) consistent with established diabetes. There was no significant difference in HbA1c levels between the four diabetic groups: diabetes (DM), diabetes treated with the LOXL2 inhibitor PXS-S2B (DM + LOXL2i), diabetes treated with telmisartan (DM + Telmi) and diabetes treated with both drugs (DM + LOXL2i + Telmi). Daily treatment with PXS-S2B over the 24-week period was well tolerated with no adverse drug effects noted, in particular no histological damage in liver, heart or lung (data not shown).

**Table 1:** Clinical characteristics of mice.

n = 5–8	Ctrl	DM	DM + LOXL2i	DM + Telmi	DM + LOXL2i + Telmi
Body weight (g)	25.0 ± 0.31	21.5 ± 0.90**	21.5 ± 0.78**	23.1 ± 0.62*	22.7 ± 0.57**
Average BGL (mmol/L)	9.7 ± 0.44	20.6 ± 0.40**	23.88 ± 0.72^##^	26.2 ± 0.50^##^	24.7 ± 0.65^##^
HbA1c (%)	4.3 ± 0.05	8.1 ± 0.18**	8.4 ± 0.12**	8.7 ± 0.23**	8.9 ± 0.50**

Average fasting BGL over the course of the experiment. Body weight and HbA1c were taken at time of sacrifice. Only mice with final HbA1c of ≥7.5% were included in the subsequent data analysis. Data are expressed as mean ± SEM of 5–8 mice per group. ^*^P < 0.05 vs. Ctrl, ^**^P < 0.01 vs. Ctrl, ^##^P < 0.01 vs. DM.

### Increased renal LOXL2 expression in mice

**Figure 1:**
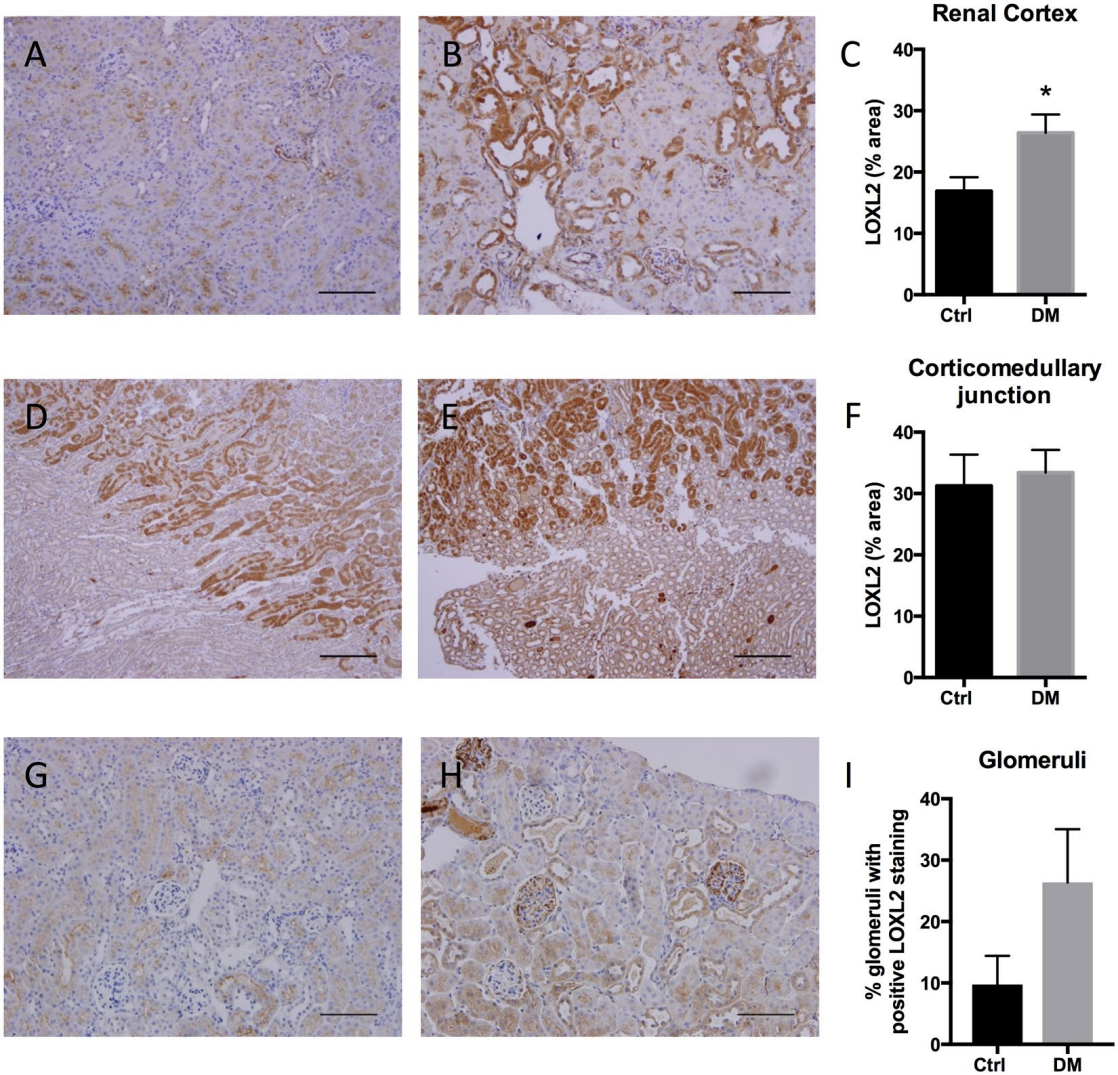
LOXL2 immunostaining in renal cortices (**A**–**C**), corticomedullary junction (**D**–**F**) and glomeruli (**G**–**I**). LOXL2 expression was significantly increased in the renal cortices of diabetic animals (DM) (**B**) vs. control (Ctrl) (**A**). The medulla had a characteristic staining pattern along the corticomedullary junction without significant difference between Ctrl (**D**) and DM (**E**). Increased numbers of glomeruli stained positive for LOXL2 in DM (**H**) vs. Ctrl (**G**). Scale bar = 200 μm. Mean ± SEM, *P < 0.05 vs. Ctrl, n = 5–7.

In order to confirm the expression of LOXL2 upon disease induction, kidneys were stained with LOXL2 antibody. LOXL2 immunohistochemistry had a distinct staining pattern with a predominantly tubular localisation. Diabetic mice had significantly increased staining in the renal cortex which was most pronounced in areas of tubular atrophy and damage (P < 0.05 vs. Ctrl, Fig. 1B). Most glomeruli of control mice did not stain for LOXL2 (Fig. 1E) whereas the diabetic mice had increased numbers of glomeruli with positive staining (Fig. 1F). Specifically mice with average fasting blood glucose >24 mmol/L had glomeruli with very strong LOXL2 immunostaining even in areas where tubular staining was minimal (Fig. 1H). Although there was strong staining along the corticomedullary junction, it did not differ between control (Fig. 1D) and diabetic animals (Fig. 1E).

### LOXL2 inhibition reduces albuminuria in diabetic mice

**Figure 2:**
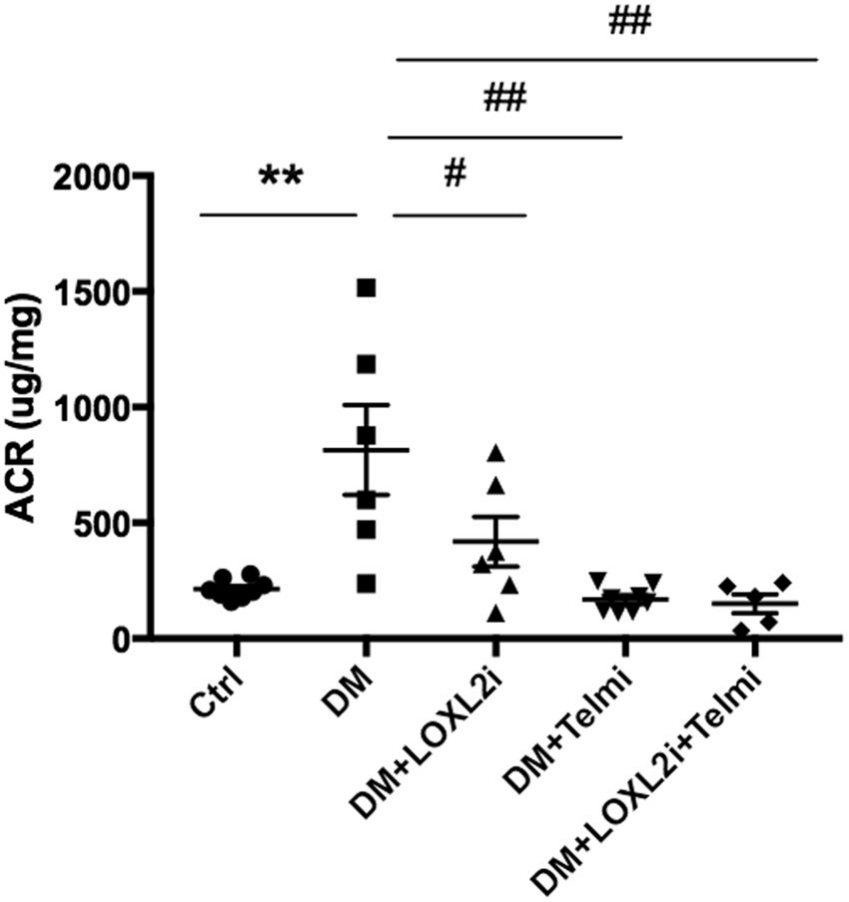
Urine albumin/creatinine ratio in control (Ctrl), diabetic (DM) and diabetic mice treated with PXS-S2B (DM + LOXL2i) and/or telmisartan (DM + Telmi, DM + LOXL2i + Telmi). Data are expressed as mean ± SEM of 5–8 mice per group. **P < 0.01 vs. Ctrl, ^#^P < 0.05 vs. DM, ^##^P < 0.01 vs. DM.

Albuminuria reflects damage to the glomerular filtration barrier and is the earliest sign of diabetic nephropathy. The excess ultrafiltered protein load then perpetuates kidney disease progression through multiple pathways including induction of chemokine and cytokine expression leading to inflammatory cell infiltration and fibrogenesis^18^. The urinary albumin to creatinine ratio obtained in the final week was nearly fourfold increased in untreated diabetic mice to 813 ± 194 μg/mg vs. 213 ± 14 μg/mg in the control group (P < 0.01 vs. Ctrl). Diabetic mice treated with PXS-S2B had significant reduction of albuminuria (418 ± 108 μg/mg, P < 0.05 vs. DM). Treatment with Telmisartan and the combination treatment (LOXL2i + Telmi) resulted in further reduction of albuminuria (168 ± 19 μg/mg, P < 0.01 vs. DM and 150 ± 41 μg/mg, P < 0.01 vs. DM respectively, Fig. 2).

### LOXL2 inhibition reduces glomerulosclerosis

**Figure 3:**
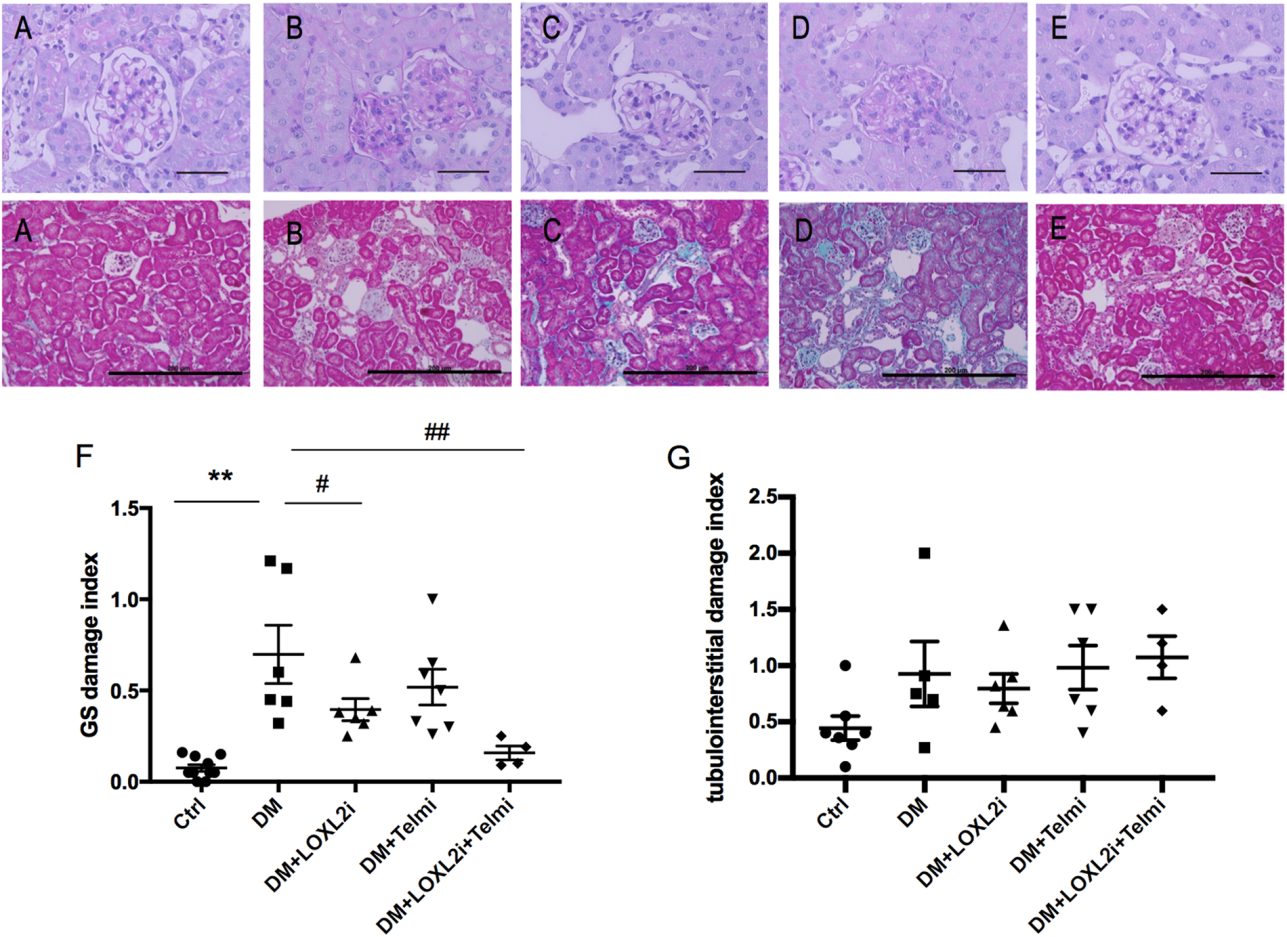
LOXL2 inhibition reduced glomerulosclerosis in diabetic mice. Top image shows representative images of PAS stained kidney sections for (**A**) Ctrl, (**B**) DM, (**C**) DM + LOXL2i, (**D**) DM + Telmi, (**E**) DM + LOXL2i + Telmi, scale bar = 50 μm. Bottom image shows Masson trichrome staining for assessment of tubulointerstial fibrosis for (**A**) Ctrl, (**B**) DM, (**C**) DM + LOXL2i, (**D**) DM + Telmi, (**E**) DM + LOXL2i + Telmi, scale bar = 200 μm (**F**) Glomerulosclerosis index score of 20 glomeruli, mean ± SEM, **P < 0.01 vs. Ctrl, ^#^P < 0.05 vs. DM, ^##^P < 0.01 vs. DM, n = 4–8. (**G**) Tubulointerstital fibrosis score of 10 non-overlapping sections per slide, mean ± SEM.

As diabetic nephropathy progresses excess ECM accumulates in the mesangium leading to glomerulosclerosis. The untreated diabetic mice (Fig. 3B) developed significant glomerulosclerosis compared to the control group (P < 0.01 vs. Ctrl, Fig. 3A). Diabetic mice treated with the LOXL2 inhibitor had significant reduction in the glomerulosclerosis score (P < 0.05 vs. DM, Fig. 3C), whereas the reduction in the telmisartan group was statistically not significant (Fig. 3D). The glomerulosclerosis index was further reduced when the treatment with the LOXL2 inhibitor and telmisartan was combined (p < 0.01, Fig. 3E). In contrast to glomerulosclerosis the diabetes induced tubulointerstitial damage was less pronounced. This was characterised by dilated tubules and flattened tubular epithelial cells with minor tubulointerstitial ECM deposition on Masson Trichrome stained paraffin sections. There was no difference seen in the treated animals in comparison to untreated diabetic mice (Fig. 3G).

### LOXL2 inhibition reduces fibronectin expression in diabetic glomeruli

**Figure 4:**
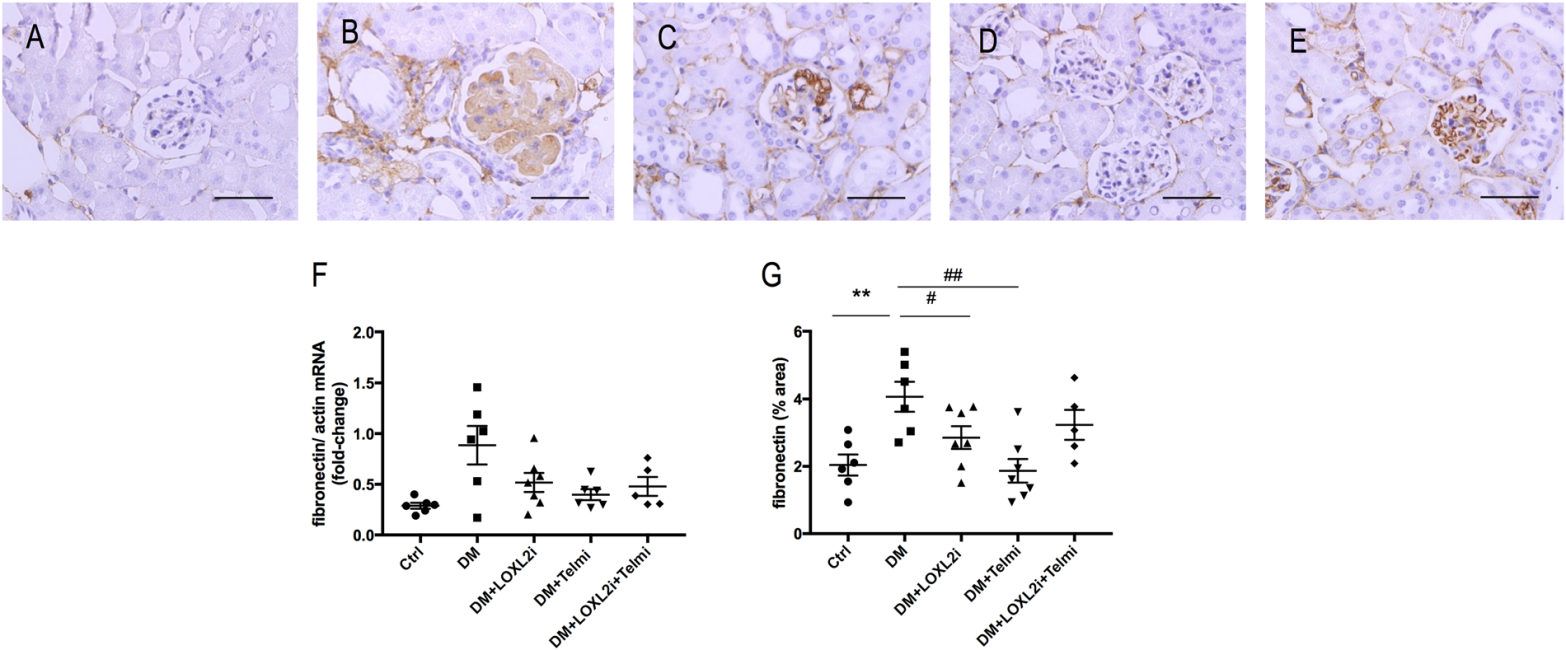
LOXL2 inhibition reduced fibronectin. Representative photographs for (**A**) Ctrl, (**B**) DM, (**C**) DM + LOXL2i, (**D**) DM + Telmi, (**E**) DM + LOXL2i + Telmi, scale bar = 50 μm, (**F**) fibronectin mRNA and (**G**) unmunohistochemistry quantification. Mean ± SEM, **P < 0.01 vs. Ctrl, ^#^P < 0.05 vs. DM, ^##^P < 0.01 vs. DM, n = 5–7.

Fibronectin, an adhesive glycoprotein activates integrins, functions as fibroblast chemoattractant and forms a scaffold for the deposition of fibrillar collagens. It is one of the first ECM proteins deposited in renal fibrosis^5^. Immunohistochemical staining for cortical fibronectin was increased in diabetic mice (P < 0.01 vs. Ctrl) and was strongly deposited in sclerosed glomeruli and to a lesser extent in the tubulointerstitial space. Diabetic mice treated with either PXS-S2B or telmisartan had significant reduction in fibronectin mRNA (P < 0.01 vs. DM and P < 0.01 vs. DM respectively) and significant reduction in fibronectin immunostaining (P < 0.05 and P < 0.01 vs. DM respectively, Fig. 4).

### LOXL2 inhibition reduces tubulointerstitial collagen I

**Figure 5:**
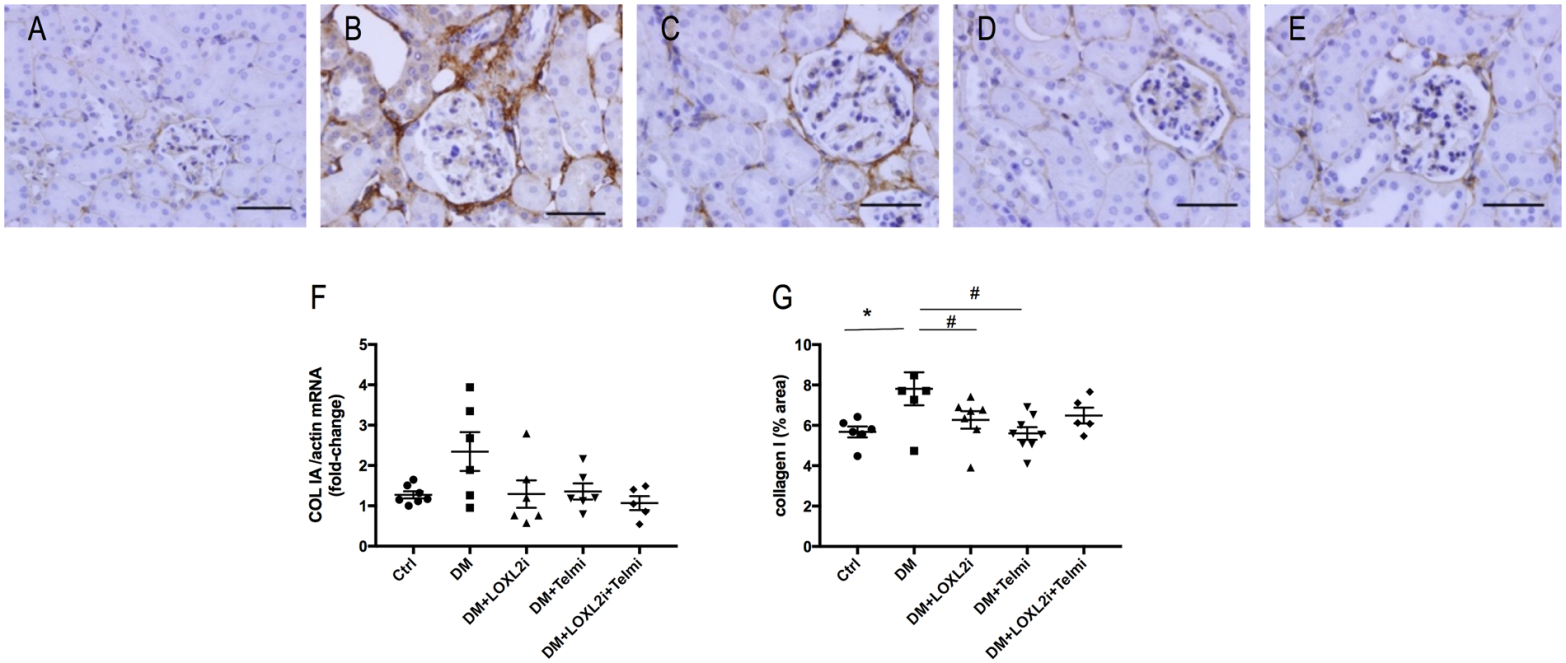
Diabetic mice had increased levels of collagen I, which were significantly reduced after treatment with the LOXL2 inhibitor. Representative images of collagen I immunostaining for (**A**) Ctrl, (**B**) DM, (**C**) DM + LOXL2i, (**D**) DM + Telmi, (**E**) DM + LOXL2i + Telmi, scale bar = 50 μm, (**F**) collagen I mRNA and (**G**) immunohistochemistry quantification. Mean ± SEM, *P < 0.05 vs. Ctrl, ^#^P < 0.05 vs. DM, n = 5–7.

**Figure 6:**
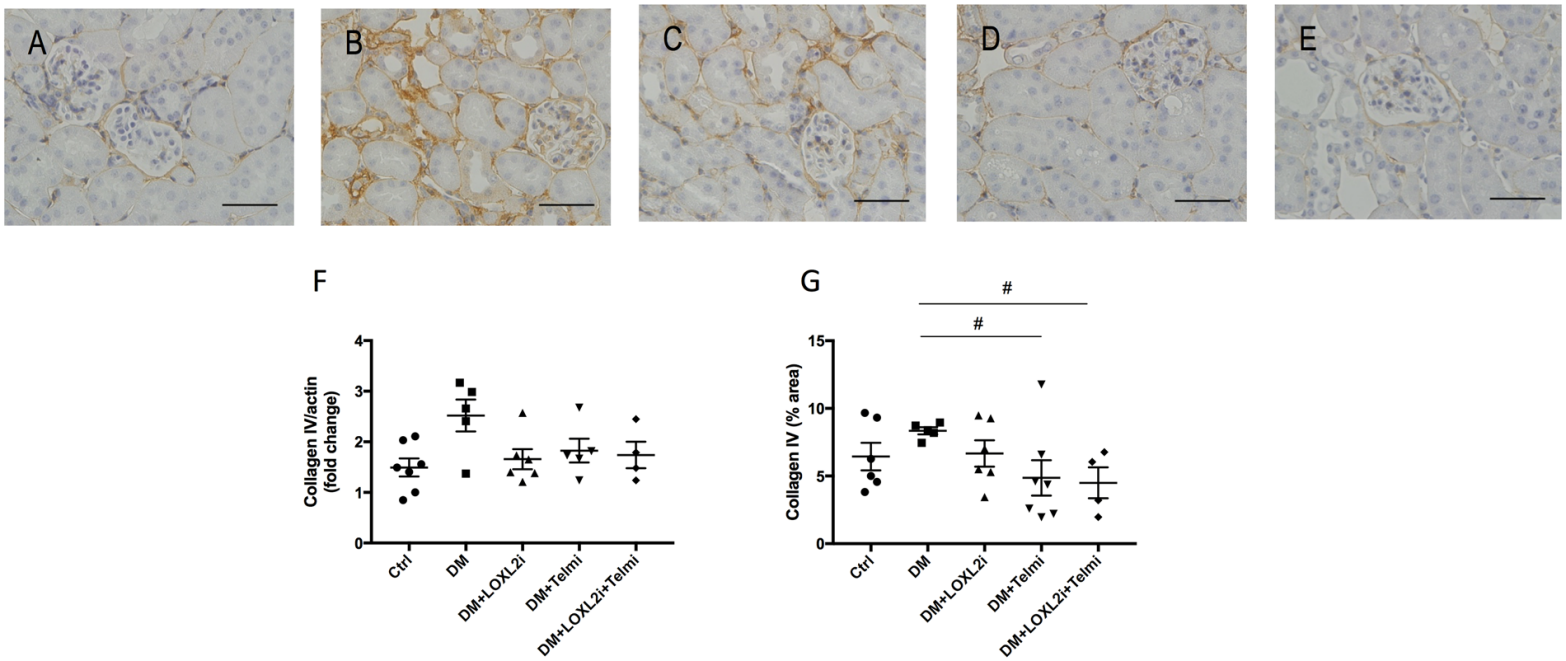
Representative images of collagen IV immunostaining for (**A**) Ctrl, (**B**) DM, (**C**) DM + LOXL2i, (**D**) DM + Telmi, (**E**) DM + LOXL2i + Telmi, scale bar = 50 μm, (**F**) collagen I mRNA and (**G**) immunohistochemistry quantification. Mean ± SEM, ^#^P < 0.05 vs. DM, n = 4–7.

Collagen I is minimally expressed in healthy kidneys but is known to increase in renal fibrosis. Similar to fibronectin, it appears in early stages of renal fibrosis^5^. Collagen I immunostaining was significantly increased in diabetic mice and was predominantly localised in the tubulointersitital space. (P < 0.05 vs. Ctrl). Treatment with PXS-S2B resulted in a significant reduction in collagen I immunostaining (P < 0.05 vs. DM). Telmisartan treatment similarly resulted in a significant reduction of collagen I immunuostaining (P < 0.05 vs. DM), which was not different from control values (Fig. 5). Collagen IV protein, the most abundant collagen in healthy kidneys and integral component of tubular and glomerular basement membranes was slighthly upregulated in diabetic kidneys although this did not reach statistical significance. There was a trend of reduction after treatment with PXS-S2B and a significant reduction in the telmisartan and in the combined treatment group (P < 0.05 vs. DM, P < 0.01 vs DM respectively, Fig. 6).

### Effect of LOXL2 inhibition on myofibroblast activation

**Figure 7:**
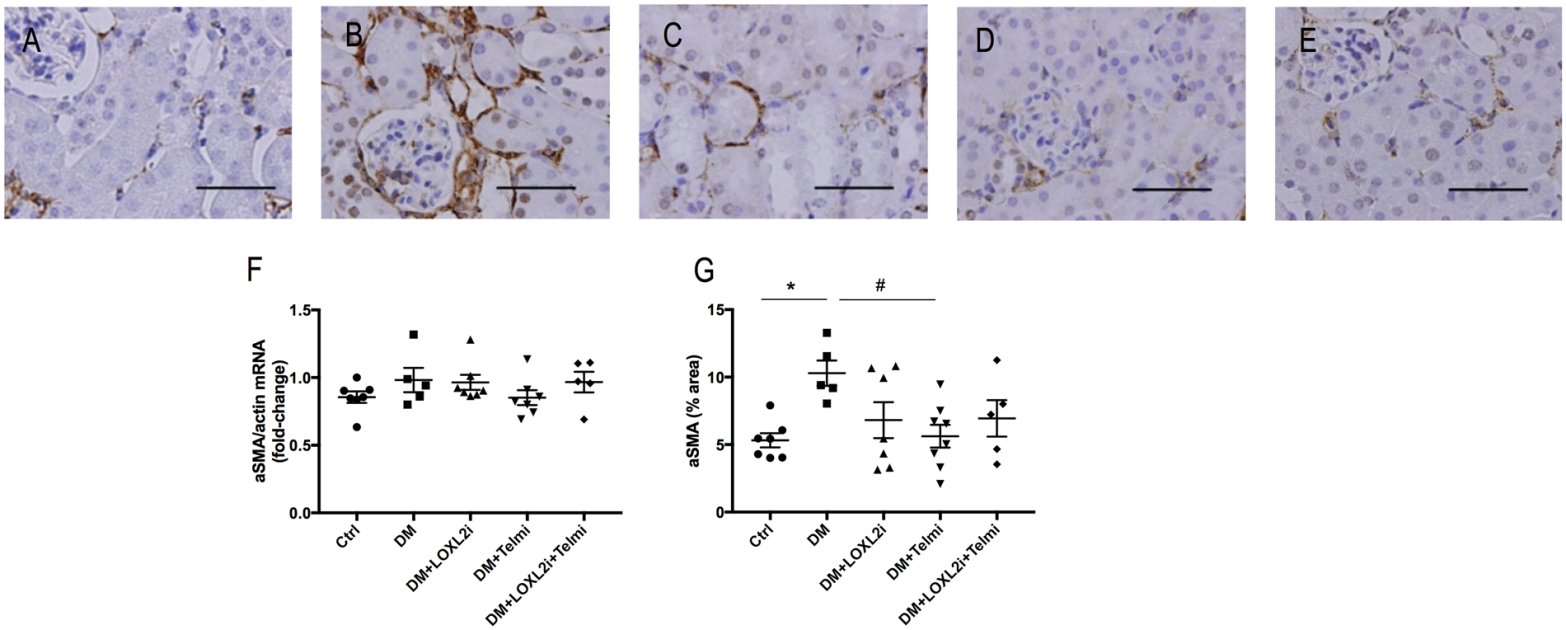
Representative images of αSMA immunostaining. (**A**) Ctrl, (**B**) DM, (**C**) DM + LOXL2i, (**D**) DM + Telmi, (**E**) DM + LOXL2i + Telmi, scale bar = 50 μm. *P < 0.05 vs. Ctrl, ^#^P < 0.05 vs. DM, n = 5–8.

De-novo expression of αSMA is a marker of myofibroblast activation, which are the principle cells responsible for the excess ECM synthesis^19^. Immunostaining of αSMA was increased in diabetic mice in our model (Fig. 7) with a trend towards reduction after LOXL2 treatment, although this did not reach statistical significance. αSMA staining was significantly reduced in the telmisartan group (P < 0.05 vs. DM) but not in the combined treatment group. The epithelial marker E-Cadherin, which is typically downregulated in EMT showed only a trend of reduction in the diabetic group not reaching statistical significance (data not shown), which suggests that EMT was not a major contributor to the myofibroblast pool in the diabetic group in our study.

## Discussion

Despite the known involvement of LOXL2 in a variety of processes that affect ECM deposition and enhance fibrosis, its role as a potential treatment target in renal fibrosis is not well studied. Using the novel, selective, small molecule LOXL2-inhibitor PXS-S2B in a mouse model of diabetic nephropathy we demonstrated both a structural and functional benefit. Diabetic animals treated with the LOXL2 inhibitor had lower albuminuria and ameliorated glomerulosclerosis with reduced cortical ECM markers. These changes were more prominently observed in the glomerular compartment than the tubulointerstitial space. We firstly, and to date, uniquely demonstrated increased levels of LOXL2 in renal cortices of mice with diabetic nephropathy. The expression pattern of LOXL2 in focal areas of strong tubular damage as well as de-novo expression in diseased glomeruli suggests its direct involvement in the renal fibrosis process. Despite the lack of difference between the diabetic and treated groups by Masson Trichrome staining, we demonstrated a significant reduction in fibronectin as well as collagen I with a trend towards lower collagen IV, suggestive of reduced tubolointerstitial fibrosis. These findings indicate that LOXL2 may be a suitable therapeutic target in diabetic nephropathy.

The induction of renal lysyl oxidases, in particular LOX and LOXL2 has been shown in several kidney disease models^10,20–22^. In Sprague Dawley rats with Adriamycin nephropathy, LOX mRNA was upregulated in glomeruli, medulla and whole kidneys early in the disease process and this preceded the development of diffuse fibrotic lesions^21^. Upregulation of LOXL2 was both observed in an experimental model of tubulointerstitial fibrosis by unilateral ureteric obstruction (UUO) and in renal biopsies of patients with chronic kidney disease of various aetiologies^10^. Higgins *et al*. found increased LOXL2 expression in the tubulointerstitial compartment of renal biopsies from patients with diabetic nephropathy, IgA nephropathy and hypertensive nephrosclerosis supporting the notion that LOXL2 may play a role in the development and progression of diabetic nephropathy and other aetiologies of renal fibrosis^10^.

The present study shows an attenuation of glomerulosclerosis and albuminuria as well as a reduction in glomerular fibronectin with the LOXL2 inhibitor treatment, indicating a protective effect in the glomerulus. This finding is supported by recent studies, which highlight the role of LOXL2 in the glomerulus. Anazco *et al*. demonstrated in microdissected mouse glomeruli that LOXL2 is the predominant lysyl oxidase and transcripts of the other lysyl oxidase family members were expressed at much lower levels. Further, it was demonstrated, that immunoreactivity in the glomerulus both localised to the extracellular and to the podocyte’s perinuclear compartment suggestive of locally and endogenously produced LOXL2 by podocytes^23^. High-throughput studies from microdissected glomeruli of patients with nephrosclerosis have also reported an increase of LOXL2^15^. By demonstrating that LOXL2 inhibition modifies ECM in diseased glomeruli we have strengthened the evidence for LOXL2 to be involved in the glomerular disease process.

Hypoxia has well been described to occur in renal fibrosis due to both tubulointerstitial and glomerular capillary rarefaction^24^. LOXL2 is induced by hypoxia via hypoxia-inducible factor-1 (HIF-1)^25^. A previous study on glomerular podocytes exposed to hypoxic conditions *in vitro* demonstrated a more than two-fold upregulation of LOXL2^26^. Hypoxia is a known fibrogenic stimulus^27^ and LOXL2 may be the connecting link in perpetuating a vicious cycle of microvascular rarefaction, hypoxia and increased ECM synthesis in the glomerulus. The amelioration of glomerulosclerosis and proteinuria in this model by selective LOXL2 inhibition with PXS-S2B appears to be an indicator that this vicious cycle can be successfully interrupted.

Fibronectin was predominantly localized in the glomerulus in our study and was reduced after treatment with PXS-S2B. Previous studies lend support for the involvement of lysyl oxidases in transcriptional regulation of non-collagen ECM proteins such as fibronectin. *In vitro* stimulation of cancer-associated fibroblasts with recombinant LOXL2 significantly increased the expression of fibronectin mRNA^8^. These stimulated fibroblast cultures also demonstrated increased levels of LOXL2 transcripts suggestive of a positive feed-forward loop to further enhance the profibrotic stimulus. Silencing of LOXL2 *both in vitro* and *in vivo* decreased fibronectin mRNA^28,29^ without affecting the expression of EMT transcription factors Snail and E-Cadherin^29^.

Additional studies have shown that LOX forms tight complexes with fibronectin, although fibronectin is not a direct substrate of LOX’s catalytic domain^30^. It is becoming more apparent that lysyl oxidases have biological roles that go beyond collagen cross-linking however the exact mechanisms by which lysyl oxidases modulate fibronectin expression remain to be shown.

In contrast to the glomerular compartment, the treatment effect was less obvious in the tubulointerstitial compartment in our study. We observed a reduction in tubulointerstitial collagen I, which is deposited in early stages of fibrosis. However, we were unable to identify a significant difference in the tubulointerstitial fibrosis score. The tubulointerstitial damage was mainly characterised by tubular atrophy while excess ECM accumulation in the diabetic groups was less pronounced. Although the eNOS−/− mouse is to date one of the most acceptable rodent models for diabetic nephropathy, the severity of tubulointerstitial fibrosis does not fully resemble that of an advanced human diabetic nephropathy^17^. It is well accepted that the genetic background is important in determining the degree of tubulointerstitial changes^31^. The C57BL/6 strain which was the only available genetic background strain for eNOS−/− mice at the outset of our study is known to be relatively resistant to develop diabetic nephropathy whereas mice on a C57BLKS background are more nephropathy-sensitive and also demonstrate greater tubulointerstitial fibrosis^32^. It is conceivable that the beneficial effect of PXS-S2B on ECM modification and tubulointerstitial matrix deposition was underestimated in our model and that the actual effect in advanced human disease, where tubulointerstitial fibrosis corresponds to decline in GFR is far greater than demonstrated. Further studies in chronic disease models with more marked tubulointerstitial fibrosis may be of value.

Myofibroblasts are the key effector cells that synthesise and secrete ECM proteins. The transient recruitment and activation of myofibroblasts represents a normal repair response to tissue injury however the persistence of myofibroblasts at sites of tissue injury is a hallmark in human fibrotic diseases^33^. The diabetic mice in this model had significantly increased numbers of activated myofibroblasts as evidenced by the elevated levels of renal αSMA. Following treatment with PXS-S2B there was a trend towards reduction in αSMA, although this did not reach statistical significance and is likely a consequence of the small sample size. EMT of injured tubular epithelial cells has been recognised to play a role in diabetic nephropathy and renal fibrosis in general, however the extent by which it contributes to the pool of activated myofibroblasts in the kidney is still a topic of intense debate. Although studies in cancer biology have demonstrated that LOXL2 is able to regulate EMT via stabilisation of the EMT transcription factor Snail^9,34^, we found no significant reduction in E-Cadherin, which as a primary downstream target of Snail is generally repressed in EMT. This finding substantiates the notion that EMT did not play a significant role in the activation of myofibroblasts in this model which is in accordance with recent fate mapping studies that have demonstrated only a small percentage of myofibroblasts are truly derived via a fully executed EMT^35^.

Previous studies have shown that physical stimuli specifically the matrix stiffness are known to play a role in the activation of myofibroblasts^36^. It has been demonstrated that fibrotic ECM activates fibroblasts to pathologically remodel the ECM via a positive feedback loop^6^. Moreover TGFβ is unable to induce myofibroblast activation in the absence of a stiff matrix^37^. We hypothesise that a disruption of cross-linking will invariably reduce matrix stiffness and this may reduce the activation of myofibroblasts.

Despite recent advances, the number of studies investigating the role of lysyl oxidases in the kidney is scarce. More knowledge has been gained from observations in other fibrotic tissues. LOXL2, in particular was implicated to play a key role in the development of fibrosis in liver^13,14^ and lung^12,13^ as well as in cancer progression where a fibrotic microenvironment facilitates motility and invasion^38^. Barry-Hamilton *et al*. detected increased LOXL2 at the fibrotic disease interface in human liver fibrosis and idiopathic pulmonary fibrosis. Treatment with a monoclonal LOXL2 antibody (AB0024) in murine models of liver and lung fibrosis resulted in amelioration of histological fibrosis scores, substantial reduction in collagen and αSMA positive fibroblasts^13^. Compared to LOXL2 inhibition, treatment with a monoclonal antibody targeting specifically LOX did not alter liver fibrosis scores^13^, suggesting LOXL2 to be the superior target of antifibrotic therapy in these organs.

Taken together the findings obtained from this study indicate an upregulation of LOXL2 in a mouse model of diabetic nephropathy and beneficial effect of LOXL2 inhibition on the structure and barrier function of the glomerulus. The inhibition of LOXL2 with a novel small molecule LOXL2 inhibitior was well tolerated and lead to the amelioration of glomerulosclerosis and proteinuria. The known multifaceted action of LOXL2 on ECM synthesis involving cross-linking of collagens, myofibroblast activation and stimulation of increased matrix products makes it a promising target in diabetic kidney fibrosis and possibly renal fibrosis of other aetiologies.

## Methods

### Animal Model

Male eNOS knockout mice (eNOS^−/−^) on C57BL/6 background were purchased from Jackson laboratory, USA. Mice were housed in single cages at 22 ± 1 °C with a 12/12 hr light dark cycle with free access to standard chow and drinking water. Diabetes mellitus was induced by intraperitoneal streptozotocin (STZ) injection (55 mg/kg diluted in sterile 10 mM citrate buffer, pH4.5 daily for 5 consecutive days) at 6–9 weeks of age. A group of mice receiving citrate buffer alone served as the non-diabetic controls. Blood glucose level (BGL) was determined by tail vein blood collection using an Accu-chek glucometer (Roche Diagnostics) after a 6 hour daytime fast. Mice with BGL >16 mmol/L two weeks post STZ injection were considered diabetic. Fasting BGLs were tested at least fortnightly. Long lasting insulin was administered as required (Insulin Glargine, Sanofi Aventis, Australia) if blood glucose levels exceeded 28 mmol/L and fasting glucose was again checked within one week^39^. The study was approved by the Animal Research Ethics Committee of the Royal North Shore Hospital (protocol number 1309-003 A) and was carried out according to the Australian Code of Practice for the Care and Use of Animals for Scientific Purposes. Animals were anaesthetised using short inhalational anaesthesia with 2% isoflurane for minor procedures and were euthanised under 2% isoflurane anaesthesia using cardiac puncture terminally.

### Experimental Design

PXS-S2B, a small-molecule selective LOXL2 inhibitor (kindly provided by Pharmaxis Ltd., Frenchs Forest, Australia) was administered by daily oral gavage (10 mg/kg) through a flexible plastic gavage tube (Instech laboratories, USA). Telmisartan (Santa Cruz, CA, USA) was mixed in drinking water (2 mg/kg/d, pH7.4) as a comparative limb of current best practice. The dose chosen is not known to have any blood pressure lowering effect^40^. Animals were divided into the following groups: 1) Control (Ctrl), 2) Diabetic (DM), 3) Diabetic receiving the LOXL2 inhibitor (DM + LOXL2i), 4) Diabetic receiving Telmisartan (DM + Telmi), 5) Diabetic receiving the LOXL2 inhibitor and Telmisartan (DM + Telmi + LOXL2i). Treatment was carried out for 24 weeks from diagnosis of diabetes. At the time of culling, kidneys were perfused in ice-cold PBS before harvest and snap frozen in liquid nitrogen or fixed in 10% neutral buffered formalin for 24 hours prior paraffin embedding.

### Compound Characteristics

PXS-S2B is a hallallylamine-derived, mechanism-based inhibitor amine oxidase inhibitor with high oral bioavailability. It distributes well into tissues and forms PXS-S2A, which has high selectivity for LOXL2 over LOX. It is characterised by good plasma stability and moderate plasma protein binding. The compound characteristics have been described in detail previously^16^.

### Physiological Parameters

Body weight was obtained monthly. Urine was collected via a 24 hour collection in metabolic cages. Urine albumin was measured by ELISA (Albuwell, Exocell Inc., USA). Urine creatinine was measured using a picric acid method (Creatinine Companion, Exocell Inc, USA). The HbA1c from a final blood collection at time of culling was determined using a DCA Vantage analyzer (Siemens Healthcare, Bayswater, VIC, Australia) according to the manufacturer’s instructions.

### Histology

Paraffin embedded kidneys sections (2 μm) were stained with Masson’s Trichrome and Periodic Acid Schiff (PAS). Assessment of glomerulosclerosis and tubulointerstitial fibrosis was done by two examiners independently. Twenty non-overlapping fields were captured under an Olympus photo light microscope linked to a Leica DFC 480 digital camera. A semiquantitative glomerulosclerosis index (GSI) score was graded on a scale of zero to four (0_normal; 1_involvement of <25% of the glomerulus, 2_ involvement of 25–50% of the glomerulus; 3_involvement of 50–75% of the glomerulus and 4_totally sclerosed glomeruli)^41^. The whole kidney average GSI score was obtained by averaging scores from all counted glomeruli in one section. For tubulointerstitial scoring ten non-overlapping fields were assessed in a similar grading system to the GS score as previously described^42^. Paraffin sections of liver, lung and heart from control and control animals treated for 24 weeks with the LOXL2 inhibitor were stained with haematoxylin and eosin (HE) and evaluated for structural damage by an independent pathologist.

### RNA Isolation and Quantitative Real-time RT-PCR

Total RNA was extracted from whole kidney tissue using the RNeasy Plus Mini extraction kit (Qiagen Valencia, CA, USA). cDNA was synthesised using the Transcriptor First Strand cDNA synthesis kit (Roche Diagnostics, Mannheim, Germany). Predesigned primers (Sigma-Aldrich, NSW Australia) for fibronectin, collagen I and IV, αSMA, E-Cadherin and actin are listed in Table 2. Quantitative real-time PCR was performed with SensiMix SYBR hiRox (Bioline, NSW Australia) on the AB7900 machine (Applied Biosystems, Australia). Gene expression is presented as fold-change compared with control after normalisation to the housekeeping gene actin.

**Table 2:** PCR primer sequences.

Target	Forward (5′-3′)	Reverse (5′-3′)
LOXL2	ATTAACCCCAACTATGAAGTG	CTGTCTCCTCACTGAAGGCTC
Fibronectin	ACAGAAATGACCATTGAAGG	TGTCTGGAGAAAGGTTGATT
COL1A1	CATGTTCAGCTTTGTGGACCT	GCAGCTGACTTCAGGGATGT
Col IV	TTAAAGGACTCCAGGGACCAC	CCCACTGAGCCCTGTCACAC
aSMA	ATAGGTGGTTTCGTGGATGC	ACTCTCTTCCAGCCATCTTTCA
E-Cadherin	CAAAGTGACGCTGAAGTCCA	TACACGCTGGGAAACATGAG
β-Actin	CTAAGGCCAACCGTGAAAAG	ACCAGAGGCATACAGGGACA

### Immunohistochemistry

Paraffin-embedded kidney sections (4 micron) were dewaxed in xylene and rehydrated in graded concentrations of ethanol before heat epitope retrieval in 10 mM citrate buffer, pH6. Endogenous peroxidase activity was blocked by incubation in 0.3% hydrogen peroxide. After a 10 min pre-incubation with 10% protein block (Dako, CA) the sections were incubated overnight at 4 °C with primary antibodies against LOXL2 (1:500 dilution, sc-66950, Santa Cruz, USA), fibronectin (dilution 1:1000, ab45688, Abcam, Cambridge, UK), collagen I (dilution 1:500, ab34710, Abcam), collagen IV (dilution 1:500, ab6586, Abcam), αSMA (dilution 1:100, A2547, Sigma Aldrich, USA). The sections were developed with 3,3′-diaminobenzidine chromogen (Dako, CA, USA) after incubation with the respective horseradish-peroxidase (HRP) tagged secondary antibodies (Dako) and then counterstained with haematoxilin. The quantification was performed by capturing 10–12 non-overlapping fields of renal cortex from stained sections at 200× magnification with a Leica microscope linked to a DFC 480 digital camera (Leica, Wetzlar, Germany). The percentage of stained area relative to the whole area in each field was assessed with image J software (Java-based software program, National Institutes of Health).

### Statistical Analysis

Data were analyzed with Prism software V 6.0 (GraphPad, La Jolla, CA) using ANOVA with Bonferroni comparison or Mann-Whitney U for non-normally distributed data and expressed as mean ± SEM. *P*-values less than 0.05 were considered to represent statistical significance.

### Data Availability

The datasets generated and analysed during the study are available from the corresponding author on reasonable request.
